# Exploring the Diversity and Novelty of Toxin Genes in *Naja sumatrana*, the Equatorial Spitting Cobra from Malaysia through *De Novo* Venom-Gland Transcriptomics

**DOI:** 10.3390/toxins11020104

**Published:** 2019-02-11

**Authors:** Ho Phin Chong, Kae Yi Tan, Nget Hong Tan, Choo Hock Tan

**Affiliations:** 1 Venom Research and Toxicology Laboratory, Department of Pharmacology, Faculty of Medicine, University of Malaya, Kuala Lumpur 50603, Malaysia; schp_182@hotmail.com; 2 Protein and Interactomics Laboratory, Department of Molecular Medicine, Faculty of Medicine, University of Malaya, Kuala Lumpur 50603, Malaysia; tanngethong@yahoo.com.sg

**Keywords:** venom-gland transcriptomics, *Naja sumatrana*, three-finger toxins, toxin genes

## Abstract

The equatorial spitting cobra, *Naja sumatrana*, is a distinct species of medically important venomous snakes, listed as WHO Category 1 in Southeast Asia. The diversity of its venom genes has not been comprehensively examined, although a few toxin sequences annotated to *Naja sputatrix* were reported previously through cloning studies. To investigate this species venom genes’ diversity, *de novo* venom-gland transcriptomics of *N. sumatrana* from West Malaysia was conducted using next-generation sequencing technology. Genes encoding toxins represented only 60 of the 55,396 transcripts, but were highly expressed, contributing to 79.22% of total gene expression (by total FPKM) in the venom-glands. The toxin transcripts belong to 21 families, and 29 transcripts were further identified as full-length. Three-finger toxins (3FTx) composed of long, short, and non-conventional groups, constituted the majority of toxin transcripts (91.11% of total toxin FPKM), followed by phospholipase A_2_ (PLA_2_, 7.42%)—which are putatively pro-inflammatory and cytotoxic. The remaining transcripts in the 19 families were expressed at extremely low levels. Presumably, these toxins were associated with ancillary functions. Our findings unveil the diverse toxin genes unique to *N. sumatrana*, and provide insights into the pathophysiology of *N. sumatrana* envenoming.

## 1. Introduction

Venom is a sophisticated and versatile weapon harnessed by venomous snakes for prey capture, and defense [1,2]. As the fundamental purpose of snake venom is predation, venom gene adaption is strongly driven by diet [3,4]. Over time, the choice and selection of prey may differ due to multiple factors, e.g., geographical and sexual variations, as well as ontogenic shifts [5,6,7,8,9]. Variations in snake venom composition has ramifications on snakebite management, as it may lead to unexpected clinical manifestations of envenomation. Moreover, variation in venom protein antigenicity can result in suboptimal antivenom efficacy or even treatment failure [10,11,12]. This is particularly relevant to snakes that are widely distributed such as cobras in Asia. Cobra venom compositions have been shown to vary widely between and within species, which can complicate the use of paraspecific antivenom, a common practice in many countries due to the unavailability or limited supply of species-specific antivenom [13,14]. 

In Southeast Asia, there are at least six to seven distinct cobra (*Naja*) species (http://reptile-database.reptarium.cz/) which can cause fatal and disabling envenomation. These include the equatorial spitting cobra, *Naja sumatrana*, a WHO Category 1 medically important venomous snakes [15]. This species is widely distributed throughout Peninsular Malaya (including West Malaysia and Singapore), Sumatra, southern part of Thailand, and part of Borneo Island [16]. The species was previously classified under a larger taxon, *Naja naja sputatrix*, and locally known as “Malayan spitting cobra”, without a clear distinction from other potentially different spitting cobras in the region such as those from the Indochina and the Java Island (Indonesia) [16]. 

The current systematics of Asiatic cobras have substantially resolved the taxonomic confusion pertaining to the taxon “*Naja naja sputatrix*”. In this context, three distinct spitting cobra species are recognized: *Naja sumatrana* (equatorial spitting cobra), *Naja siamensis* (Indochinese spitting cobra), and *Naja sputatrix* (Javan spitting cobra) [16]. Envenoming by these species often result in systemic neuromuscular paralysis and extensive local tissue necrosis. Local tissue necrosis can potentially lead to amputation and crippling disabilities in surviving victims [17]. The spitting cobras’ ability to spray venom, a well evolved defense strategy in these species, can cause venom ophthalmia and blindness [17,18,19]. Previous studies have shown that cobra venoms consist mainly of three-finger toxins and phospholipases A_2_, while their relative abundances, subtypes, and antigenicity can vary substantially between and within species [20,21]. Comprehensive understanding of the venom profile of individual species is very much dependent on the availability of species-specific databases with respect to venom gene sequences. This can be efficiently accomplished via high-throughput gene sequencing of tissues derived from authenticated specimens [6,22,23]. The present study aims to investigate the *de novo* venom-gland transcriptome of *Naja sumatrana* from Malaysia, to obtain a comprehensive profile of its venom genes using next-generation sequencing (NGS) technology. The findings will shed light on the diversity of venom genes specific to this unique species of spitting cobra in Malaysia, and provide deeper insights into the correlation of toxin composition and pathophysiology of cobra envenomation. In addition, the information obtained can be used to validate several toxin sequences annotated to “*Naja naja sputatrix*” available in the open database. These toxin sequences were reported from cloning studies in the late 1990’s and early 2000’s [24,25,26,27,28]. Unfortunately, the authenticity of the snake species used in the early days was difficult to be ascertained as the work was carried out at a time when confusion perhaps still existed in the systematics of spitting cobras. 

## 2. Results and Discussion

### 2.1. Sequencing Output Statistics and De Novo Transcriptome Assembly

**Figure 1:**
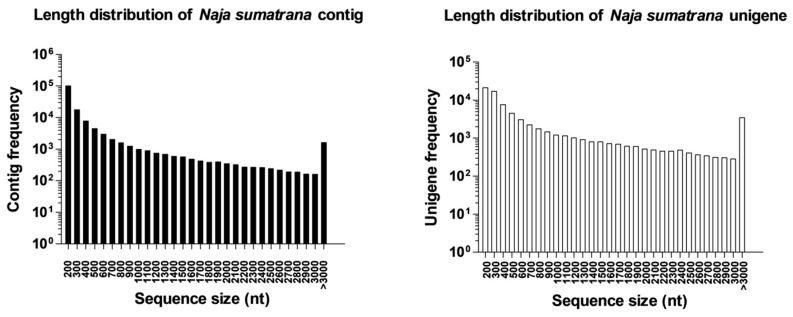
Length distribution of contigs (left) and Unigenes (right) obtained following *de novo* transcriptome assembly.

Sequencing of the cDNA libraries yielded a total of 46,878,172 clean reads for the Malaysian *Naja sumatrana* (herein NS-M) venom-gland transcriptome (Table 1). *De novo* assembly using the Trinity program created 148,475 contigs (N50 = 652) that were connected to form 75,387 Unigenes (N50 = 1702), with the length distribution shown in Figure 1. The high Q20 percentage of 97.94% indicated that the *de novo* assembly of NS-M venom-gland transcriptome was successful and of high quality. The 75,387 Unigenes assembled underwent filtering based on FPKM (fragments per kilobase per million) values, where transcripts with less than 1 FPKM mapped reads were removed. This reduced the number of Unigenes to 55,386. Following BLASTx alignment, the Unigenes—herein referred as transcripts—were assigned to three categories: (a) “unidentified” (transcripts whose gene/protein identities could not be identified during BLASTx alignment); (b) “non-toxin” (transcripts that encoded proteins which have no putative toxin role); and (c) “toxin” (transcripts that encoded known and putative toxins). The details of the results are summarized in Table 1.

### 2.2. Categorization of Transcripts and Gene Expression

**Figure 2:**
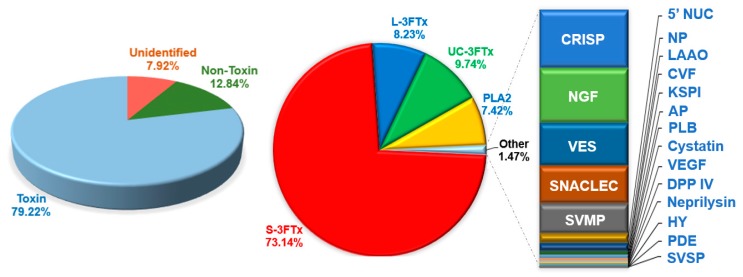
Relative abundance of transcripts (in percentage of FPKM) expressed in the venom glands of Malaysian *Naja sumatrana*. Toxin transcripts dominate the overall expression (79.22%). Within toxin transcripts, three-finger toxin (3FTx) is the most abundantly expressed toxin family in the venom glands (91.11% of the toxin FPKM). Of these, the short three-finger toxins (S-3FTx) constitute 73.14%, whereas the long three-finger toxins (L-3FTx) constitute 8.23%. Non-conventional three-finger toxins (NC-3FTx) and phospholipases A_2_ (PLA_2_) constitute 9.74% and 7.42%, respectively. Abbreviations: S-3FTx, short three-finger toxin; L-3FTx, long three-finger toxin; UC-3FTx, non-conventional three-finger toxin; PLA_2_, phospholipase A_2_; CRISP, cysteine-rich secretory protein; NGF, nerve growth factor; VES, vespryn; SNACLEC, snake venom C-type lectin/lectin-like protein; SVMP, snake venom metalloproteinase; 5’NUC, 5’ nucleotidase; NP, natriuretic peptide; LAAO, L-amino acid oxidase; CVF, cobra venom factor; AP, aminopeptidase; PLB, Phospholipase B; KSPI, Kunitz-type serine protease inhibitor; VEGF, vascular endothelial growth factor; HY, hyaluronidase; DPP IV, dipeptidylpeptidase IV; PDE, phosphodiesterase; and SVSP, snake venom serine protease.

The “toxins” category consisted of transcripts that code for a great variety of toxin proteins. Although the toxin transcripts only accounted for 60 of the 55,396 transcripts obtained, they were highly expressed and contributed to 79.22% of total gene expression (by total gene FPKM) in the venom gland. Both “non-toxin” and “unidentified” groups were composed of very high numbers of genes but the gene expression levels were low, accounting for only 12.84% and 7.95%, respectively, of the total genes expressed (Figure 2). The “non-toxin” group mainly consisted of innocuous housekeeping genes, such as transcription factors, ribosomal proteins and miscellaneous proteins which are involved in cell metabolism. The expressions of toxin genes in NS-M venom glands were comparable to those reported for the Thai *Naja kaouthia* (82%) [6], Chinese *Naja atra* (70.24%) and *Bungarus multicinctus* (69.60%) [29]. However, the levels were much higher than those found in the Malaysian king cobra (*Ophiophagus hannah*; 35.30%), and the monocled cobra (*Naja kaouthia*) of Malaysia and China (41.20% and 54.42%, respectively) [6,30,31]. The variable venom gene expression could be due to inter- or intraspecific variation, or sampling times when there are differences in the snake’s physiological conditions during venom-gland harvesting. Nevertheless, collectively these findings show that toxin genes are highly expressed in venom-gland tissue, despite the limited number of toxin transcripts (Table 1).

Exceptionally high redundancies were also observed in the toxin transcripts (20,509.14 FPKM/transcript). This is in sharp contrast to that observed in the non-toxin group of transcripts (10.03 FPKM/transcript). This indicates that toxins were highly expressed in multiple isoforms within a restricted set of gene families. The high redundancy of toxin gene expression in NS-M was consistent with findings from venom-gland transcriptomic studies of the Southeast Asian *Naja kaouthia* (monocled cobra), whereby the redundancy levels were reported to be 6,300–23,000 FPKM/transcript [6]. This is also in line with the theory behind the molecular diversity of snake venom proteins, where molecular adaptation is largely driven by repeated gene duplication followed by neofunctionalization of the proteins [1,2]. 

### 2.3. Complexity of N. sumatrana Venom-Gland Transcriptome

The 60 toxin transcripts derived from NS-M venom glands were classified into 21 gene families. A total of 29 transcripts were further identified as full-length (Table 2). The three-finger toxins (3FTx) inclusive of long, short, and non-conventional groups, constituted the majority of toxin transcripts (91.11% of total toxin FPKM), followed by phospholipase A_2_ (PLA_2_, 7.42%). The remaining transcripts in 19 families were expressed at extremely low levels, each constituting less than 0.4% of total FPKM (Figure 2). These were, in descending order of expression, cysteine-rich secretory protein (CRISP), nerve growth factor (NGF), vespryn (VES), snake venom C-type lectin/lectin-like protein (SNACLEC), snake venom metalloproteinase (SVMP), 5’ nucleotidase (5’ NUC), natriuretic peptide (NP), L-amino acid oxidase (LAAO), cobra venom factor (CVF), Kunitz-type serine protease inhibitor (KSPI), aminopeptidase (AP), phospholipase B (PLB), cystatin, vascular endothelial growth factor (VEGF), dipeptidyltidase IV (DPP IV), neprilysin, hyaluronidase (HY), phosphodiesterase (PDE), and snake venom serine protease (SVSP) (Table 3).

Out of the 60 toxin transcripts, seven were annotated based on homology with sequences reported for *Naja naja sputatrix* (deposited previously in the open database). There is currently no toxin gene annotated by *N. sumatrana* in the database. The species of *Naja sumatrana* was erected and fully recognized only about 20 years ago [16]. Prior to that, toxinologists seldom distinguished between the three species of spitting cobras (*N. sumatrana*, *N. sputatrix*, and *Naja siamensis*) distributed in this region. Gene cloning studies on *N. naja sputatrix* by Jeyaseelan and colleagues [24,25,26,27,28] were based on venom glands extracted from spitting cobra(s) that appeared to originate from the Peninsular Malaya or Singapore. In view of the identical match of the sequences to *N. sumatrana* transcripts in the present study, it is very likely that the spitting cobra labeled as “*N. naja sputatrix*” previously was actually an *N. sumatrana*. The venom-gland transcriptome of NS-M hence can be used to validate the existing sequences annotated to *N. naja sputatrix* in the database.

Besides 3FTx and PLA_2_, the remaining 53 toxin transcripts were matched by at least 50% sequence similarity with toxins from other snake species of *Naja* (25 transcripts) and other genera (28 transcripts), probably because of the paucity of sequence database specific for the Southeast Asian spitting cobras.

### 2.4. Diversity of Toxin Transcripts and Major Venom Constituents

#### 2.4.1. Three-finger Toxins (3FTxs)

**Figure 3:**
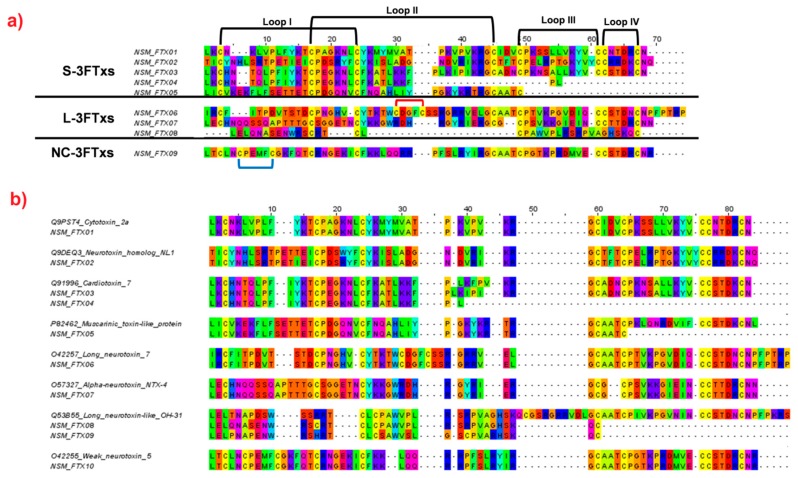
Multiple sequence alignments of the short, long, and non-conventional three-finger toxin (3FTx) transcripts of Malaysian *N. sumatrana*. (**a**) 3FTx transcripts were aligned based on the subgroups. (**b**) 3FTx transcripts were aligned and compared to annotated sequences obtained from public database. In (**a**): Black bracket represent disulfide linkages. Red and blue brackets highlight the fifth additional disulfide bridge located in the second and first loop of L-3FTx and NC-3FTx, respectively.

Three-finger toxins (3FTxs) are non-enzymatic polypeptides with 60–74 amino acids, stabilized by four or five disulfide cross-linkages [32]. The characteristic feature of 3FTx is their distinctive protein folding, containing a hydrophobic core with three extended beta-stranded loops. Its structural integrity is supported by the conserved peptide regions and four cross-linked disulfide linkages at the core [33,34,35]. The venom-gland transcriptomic results revealed a total of 10 distinct 3FTx transcripts. These transcripts altogether accounted for 91.11% of the total FPKM of all toxin transcripts. The transcripts were further categorized into short-chain (S-3FTxs; five transcripts; 73.14%), long-chain (L-3FTxs, four transcripts; 8.23%), and non-conventional (NC-3FTxs; one transcript; 9.74%) subgroups of 3FTx (Table 3). Organization of the 3FTx subtypes was based on the location and number of disulfide bonds that maintain the protein structure (Figure 3), where S-3FTxs possess four disulfide bonds, whereas L-3FTxs and UC-3FTxs carry a fifth disulfide bond at the lateral end of the second loop and first loop, respectively [32]. Among the ten 3FTx transcripts, four were annotated to *Naja sputatrix* (one cytotoxin “NSM_3FTX01”, two long neurotoxins “NSM_3FTX06; NSM_3FTX07”, one weak neurotoxin “NSM_3FTX09”). The amino acid sequences of the transcripts were found to be 100% identical to the sequences deposited as *N. naja sputatrix* toxins in the database. These four 3FTx sequences of *N. sputatrix* were derived from previous cloning and expression studies using tissues from spitting cobra(s) in Peninsular Malaya or Singapore (Q9PST4 [26]; O42257 [27]; and O57327 [25]). The 100% match with sequences obtained in the present transcriptomic study strongly suggests that the spitting cobra described as *N. n. sputatrix* or *N. sputatrix* used in earlier studies was most likely *N. sumatrana* of Malaysian origin. The other six transcripts of 3FTxs in this study were novel, showing distinct amino acid substitutions compared to the homologous sequences of other *Naja* cobra species.

It is worthy to note that the four transcripts identical to 3FTx sequences of *N. sputatrix* (whose true identity should be *N. sumatrana* as explained above) were highly expressed, with NSM_3FTX01 (identical to cytotoxin 2a) accounting for 72.93% of total toxin FPKM. This was followed by NSM_3FTX09 (weak neurotoxin 5, 9.74%), NSM_3FTX06 (long neurotoxin 7, 4.91%), and NSM_3FTX07 (α-neurotoxin NTX-4, 3.30%). The remaining 3FTx subtypes that were matched to homologous sequences of other cobra species were expressed in extremely low abundance, each constituting far less than 1% of the total toxin FPKM.

Within the S-3FTx subfamily, the transcripts coded for two cytotoxins, a short neurotoxin and a muscarinic toxin-like protein. The expression of cytotoxin genes, in particular the major subtype (NSM_3FX01), far exceeded the abundance of transcripts for short neurotoxins and muscarinic toxin-like protein. The expression of cytotoxin in the venom glands of NS-M is in agreement with findings from the corresponding proteomic study whereby cytotoxins (also known as cardiotoxins) were reported as the main 3FTx subtype group of the venom proteins (44.41%) [36]. Cytotoxins are cytolytic proteins that are associated with necrotizing effect of cobra venom; the high abundance of CTX was consistent with extensive local tissue necrosis observed clinically at the wound site of cobra bite [37,38], and during *in vitro* cytotoxicity experiments [39]. On the other hand, short neurotoxins were expressed in a much lower abundance (0.21% by total toxin FPKM), although the proteomic study reported an abundance of 3.5% of short neurotoxins [36]. One muscarinic toxin-like protein transcript was detected at an extremely low FPKM; the presence of this protein has not been reported in the venom proteome of *N. sumatrana*. Meanwhile, the presence of alpha-neurotoxins in the venom-gland transcriptome corroborates the neurotoxicity of *N. sumatrana* envenomation. Alpha-neurotoxins bind to postsynaptic nicotinic acetylcholine receptors (nAChRs) at the skeletal neuromuscular junction, resulting in systemic paralysis and rapid death in cobra envenoming [40,41]. Three transcripts (NSM_FTX06, NSM_FTX07, and NSM_FTX08) detected were similar to long neurotoxins of the L-3FTx subgroup. NSM_FTX06 and NSM_FTX07 were expressed at similar levels (4.91% and 3.30%, respectively), whilst NSM_FTX08 was expressed at a very low level (0.01%).

There was only one NC-3FTx subtype detected in the NS-M venom-gland transcriptome, annotated as weak neurotoxin 5 (NSM_FTX09). Weak toxin has not been detected in the venom proteome of *N. sumatrana* [36]. The pathophysiological role of this toxin subtype in envenomation is poorly understood. Weak neurotoxin derived from monocled cobra venom has been shown previously as an antagonist of human and rat neuronal nicotinic receptors, but it has very low lethality (median lethal dose (LD_50_) of up to 5–80 μg/g) [42] compared to the highly lethal alpha-neurotoxins that have LD_50_ values of ~0.1–0.2 μg/g in mice [11,36,43,44]. 

The current transcriptomic findings on the diversity of principal toxins *i.e.* 3FTx correlated with the characteristic toxic activities of the venom. These include post-synaptic neuromuscular paralysis and severe tissue necrosis [17,18]. The presence of multiple distinct 3FTx transcripts also implies that this toxin group is highly evolved, and the role of 3FTx diversity in predation and envenomation deserves further investigation [45]. Furthermore, the current study revealed that the sequences of alpha-neurotoxins and cytotoxins specific to *N. sumatrana* exhibit marked consensus sequences with other cobra species such as *Naja kaouthia* and *Naja atra*. 

The discrepancy between transcriptomic and proteomic expression has been shown in several other studies [6,30,46], although some studies reported conflicting findings [47,48]. The phenomenon could be attributed to a variety of factors including varying rates of mRNA expression for different toxins and post-translational modification of protein [49].

#### 2.4.2. Phospholipase A_2_ (PLA_2_)

**Figure 4:**
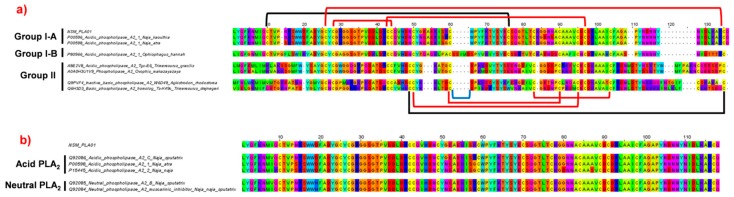
Multiple sequence alignment of phospholipase A_2_ (PLA_2_) transcript of the Malaysian *N. sumatrana*. (**a**) Multiple sequence alignment visualizing different groups of PLA_2_. (**b**) Sequence alignment of acid and neutral PLA_2_. In (**a**): Brackets in black represent disulfide bridges; red represents conservative disulfide bonds and blue represents pancreatic loop.

The secretory phospholipases A_2_ (PLA_2_) was the second most expressed toxin family of *N. sumatrana* venom gland. Two distinct PLA_2_ transcripts were detected, with the major transcript (NSM_PLA01) expressed at 7.39% of total toxin FPKM, followed by NSM_PLA02 (0.03%). The NSM_PLA01 is devoid of a pancreatic loop and is homologous with PLA_2_ of group I-A (Figure 4a). It is identical to the deposited acidic PLA_2_ sequence (UniprotKB: Q92086) (Figure 4b) reported previously for the Malayan spitting cobra labeled as *N. sputatrix* [24]. These findings again indicate that the spitting cobra specimen identified as *N. sputatrix* in the previous study was most likely *N. sumatrana* of Malaysian origin, and reaffirms the PLA_2_ sequence (UniprotKB: Q92086) specific to *N. sumatrana*. On the other hand, the other PLA_2_ transcript (NSM_PLA02) sequence was similar to a neutral PLA_2_ of the banded sea krait, *Laticauda semifasciata*.

In contrast to the high PLA_2_ content (~31% by total protein) reported in the venom proteome [36], the expression of PLA_2_ transcripts by FPKM was much lower (~7.4%). The intense transcription of 3FTx genes (~91% of all toxins) could possibly reduce transcription levels of other toxin groups at the time of tissue sampling. The transcription of different genes probably proceeds at different rates, and transcripts with low abundance may have a long half-life that in a higher amount of translated protein.

Two PLA_2_ sequences from *N. sumatrana*, *i.e.*, an acidic (Q92086) and a neutral PLA_2_ (Q92085) (previously labeled as for *N. sputatrix*), are readily accessible in the public database. These two sequences differ by only two amino acids, specifically on residues 20 and 46 (Figure 4). Substitution of the amino acids would have modified the isoelectric points (pI) of the PLA_2_ to 5.19 and 6.07, respectively, for the acidic and neutral PLA_2_s. In the present study, the acidic PLA_2_ transcript (NSM_PLA01) was 100% matched to Q92086 (acidic type), while the neutral PLA_2_ transcript (NSM_PLA02) was highly homologous to Q8JFB2, a neutral PLA_2_ (pI = 6.92) originating from *L. semifasciata*. Although a number of acidic PLA_2_ from Asian cobras and sea snakes were found to be non-lethal [11,44,50,51,52], the neutral PLA_2_ of *N. sumatrana* has been shown to be lethal in mice with an intravenous LD_50_ of 2.00 μg/g [43]. Clinically, the pathological effect of the neutral PLA_2_ is not known, but it has been shown to potentiate the toxic activity of cobra cardiotoxin/cytotoxin [53], and is hence a candidate toxin to be targeted for neutralization by antivenom. In addition, the high PLA_2_ enzymatic activity of *N. sumatrana* venom has been found to be comparable to other spitting cobras, implying that the PLA_2_ could have a local tissue effect related to venom ophthalmia [54].

#### 2.4.3. Phylogenetic Analysis of Cytotoxin and Phospholipase A_2_

**Figure 5:**
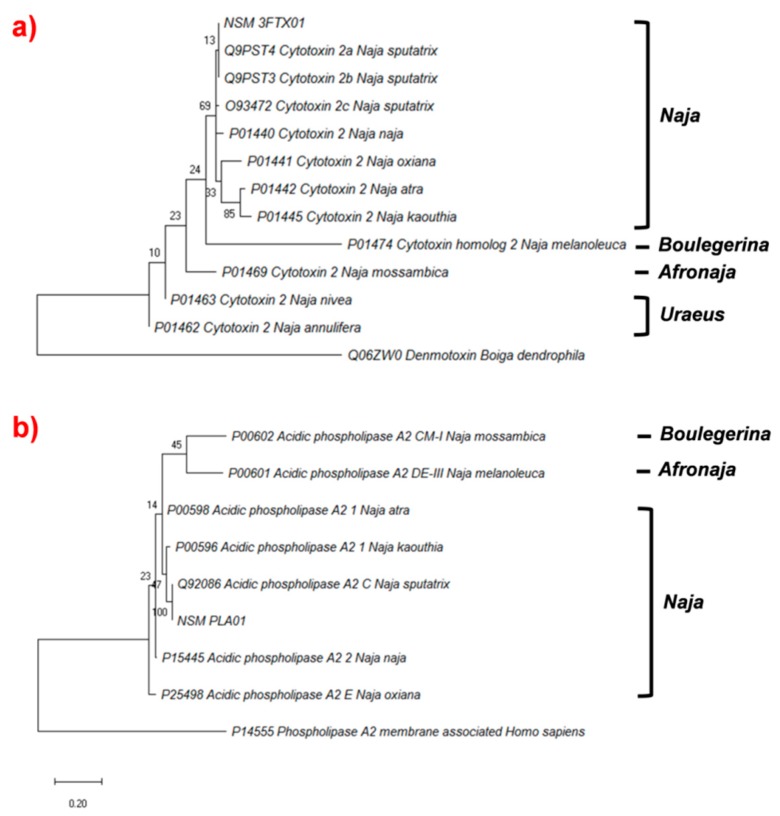
Phylogenetic trees of cytotoxin and phospholipase A_2_ transcripts of Malaysian *N. sumatrana*. The tree was constructed using MEGA X (version 10.0.5), set to Maximum likelihood method, with bootstrap value of 100. (**a**) Phylogenetic tree of NSM_FTX01 with selected cytotoxin sequences retrieved from various *Naja sp.* (UniprotKB accession numbers of proteins selected: Q9PST4; Q9PST3; O93472; P01440; P01441; P01442; P01445; P01469; P01462; P01463, and P01463). (**b**) Phylogenetic tree of NSM_PLA01 with acidic phospholipases A_2_ from various *Naja sp.* (UniprotKB accession numbers of proteins selected: Q92086; P00596; P00598; P15445; P25498; Q5G290; P00601, and P00602).

Phylogenetic analysis of cobra cytotoxins indicated that genes from the African non-spitting species (subgenus *Uraeus*) appeared to be basal (Figure 5a). The CTX 2 genes in Asiatic cobras (subgenus *Naja*) were further derived. The findings showed that sequences from the previously reported *N. sputatrix* (Q9PST3, Q9PST4) and those from *N. sumatrana* (this study) were virtually identical based on their undivided phylogenetic relationship. On the other hand, Figure 5b shows that acidic phospholipases A_2_ genes from the Asiatic cobras (subgenus *Naja*) were basal in the phylogenetic tree, but a similar unbranched relationship was again observed between the acidic PLA_2_ sequences from *N. sputatrix* (Q92086) and *N. sumatrana* (this study). These findings further support that *N. sumatrana* is phylogenetically diverged from other Afro-Asian cobras, and that toxin sequences previously reported as from *N. sputatrix* most likely originated from *N. sumatrana* in Malaysia. The phylogenetic relationship of the toxins between *N. sumatrana* and genuine *N. sputatrix* (Javan origin) remains to be further investigated, as little is known about the venom-gland transcriptome of the Javan *N. sputatrix*.

### 2.5. Low Abundance Transcripts

#### 2.5.1. Transcript Expression of Toxin Families Previously Reported in Venom Proteome 

**Figure 6:**
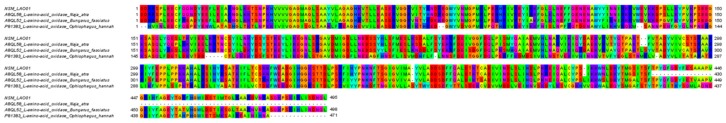
Multiple sequence alignment of L-amino acid oxidase (LAAO) transcript of Malaysian *Naja sumatrana*.

Other toxins transcripts were expressed at low abundance (below 0.4% of total FPKM) in NS-M venom glands. Toxin families whose proteins have been previously reported in the venom proteome include L-amino acid oxidase (LAAO), cobra venom factor (CVF), nerve growth factors (NGF), and vespryn. In the present study, one fully sequenced LAAO transcript was obtained (NSM_LAO01). The sequence contained the three well conserved domains of LAAO and was found to be highly homologous with LAAO sequences from different lineages including other cobra species (Figure 6). This enzyme was of low abundance (at transcript and protein levels) and it showed minimal sequence mutations, consistent with its rather conserved biological function in snake venoms [55,56]. LAAOs are flavoenzymes, found virtually in all front-fanged snake venoms including those of sea snakes [10,50,56,57], which were once thought to be devoid of LAAO. The enzymatic activity catalyzes oxidative deamination of L-amino acids to form alpha-keto acid, ammonia, and hydrogen peroxide; the pharmacological activities are diverse but in cobra venoms it is likely related to anti-microbial and digestive purposes. 

**Figure 7:**
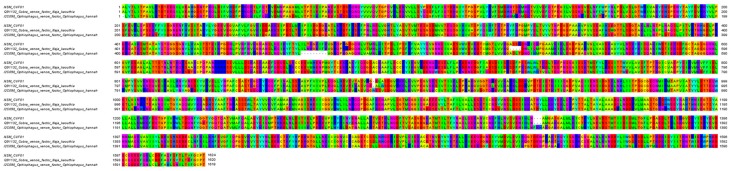
Multiple sequence alignments of cobra venom factor (CVF) transcript of Malaysian *Naja sumatrana*.

**Figure 8:**
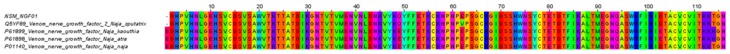
Multiple sequence alignment of nerve growth factor (NGF) transcript of Malaysian *Naja sumatrana*.

Three CVF (cobra venom factor) transcripts were uncovered in the NS-M venom-gland transcriptome. Amongst the three, a full-length sequence (NSM_CVF01) was obtained. NSM_CVF01 was almost indistinguishable from the CVF of *N. kaouthia* (UniprotKB: Q91132), with high homology at 99.82% (Figure 7). It has been suggested that the role of CVF is related to activation of complement system in the prey to increase vascular permeability and blood flow at the bite site [58]. Hence in envenoming, CVF action can promote rapid dissemination of toxin components into the blood circulation of the victim. This effect may also be facilitated by other protein components in the venom, such as nerve growth factor (NGF) that has been shown to cause plasma extravasation and histamine release, rendering the vascular tissue “leaky” to accelerate toxin diffusion [59,60]. Two transcripts of nerve growth factors (NSM_NGF01 and NSM_NGF02) were uncovered in this study with low expression (0.31% of total gene FPKM). This is consistent with the low abundance of NGF protein in the venom proteome [36]. On the other hand, the transcript NSM_NGF01 was highly homologous to the NGF reported for *N. sputatrix* (UniprotKB: Q5YF89) (Figure 8). The NGF reported has been shown to prevent metalloproteinase autodigestion of venom proteins, which is important for the stability of snake venom [59].

A full-length transcript (NSM_VES01) identical to thaicobrin (a vespryn protein) was also revealed in the current study. Similar to NGF, gene expression of vespryn was very low (0.25% of total toxin FPKM). This transcript was 93% homologous to ohanin of king cobra (*Ophiophagus hannah*), which has been shown to induce hyperalgesia and hypolocomotion in mice [61,62]. The vespryn in *N. sumatrana* venom may possess similar pharmacological actions and aid in prey immobilization.

#### 2.5.2. Transcript Expression of Toxin Families Not Reported in Venom Proteome of *N. sumatrana*

Next generation sequencing is an efficient technology in snake venomic studies for exploring the diversity and novelty of toxin genes, even though the gene products (toxin proteins) may not be detected at proteomic level. The current transcriptomic study has successfully identified 15 distinct protein families not previously reported in *N. sumatrana* venom proteome [36]; these were CRISP, SNACLEC, SVMP, 5’NUC, NP, KSPI, AP, PLB, cystatin, VEGF, DPP IV, neprilysin, HY, PDE, and SVSP. These proteins were probably expressed at extremely low abundance levels, which were not detected by the mass spectrometry in the previous proteomic study. Notably, some proteins such as SNACLEC, SVSP, and SVMP were typically present abundantly in viperid snake venoms, contributing to hemotoxic effects such as thrombocytopenia, venom-induced consumptive coagulopathy, and hemorrhage [63]. However, none of these clinical effects have been reported in *N. sumatrana* envenomation. These proteins, if expressed, likely serve ancillary functions that aid in predation and digestion of prey.

It must be emphasized that the present transcriptomic study was based on the venom glands of a single adult specimen. The use of a single specimen is common in venom-gland transcriptomic studies due to the scarcity of specimen, and the need to reduce the number of animals sacrificed. This approach limits the interpretation of whether the transcriptional pattern observed is unique to an individual snake, or if it is representative of the entire species [6,22,29,30,45]. Factors such as ontogenic, sexual, and geographical differences may result in inter-individual variation of gene transcription [6,22]. Nonetheless, the transcriptomic data from the current study provide insights into the complexity of venom genes specific to the species *N. sumatrana*. Furthermore, the sequence database established is valuable for molecular and evolutionary characterization of snake toxins in the future.

## 3. Conclusions

The *de novo* venom-gland transcriptomic analysis revealed a unique profile of venom genes in *N. sumatrana*. The distribution and expression levels of the principal toxin components, i.e., the three-finger toxins and phospholipases A_2_, provide deep insights into the toxic syndrome and pathophysiology of *N. sumatrana* envenomation. Notably, highly expressed cytotoxins, whose action may be synergistically enhanced by phospholipases A_2_, are associated with local tissue necrosis and venom ophthalmia. The alpha-neurotoxins, composed of both short-chain and long-chain subtypes, correlate with post-synaptic neuromuscular paralysis observed in envenomation. Furthermore, full-length toxin sequences obtained from the study provide a reference for validating the origin of sequences previously deposited under the name of *Naja sputatrix*. These findings also consolidate the knowledgebase of venom genes of this medically important cobra species of Malaysian origin.

## 4. Materials and Methods

### 4.1. Preparation of Snake Venom-Gland Tissue

The adult Malaysian *N. sumatrana* (NS-M) snake was captured in the south-west region of Peninsular Malaysia. The snake was milked for venom and allowed to rest for four days to maximize transcription [64]. The venom glands were promptly removed after euthanasia and sectioned into dimensions of <5 × 5 mm. The slices were preserved in RNAlater^®^ solution (Ambion, Texas, USA), at 4 °C overnight for the efficient penetration of solution into the sample before transferring to storage at −80 °C until further use. The study was conducted in accordance with the experimental protocol approved by the Institutional Animal Use and Care Committee (IACUC) of University of Malaya, Malaysia (code: #2013-11-12/PHAR/R/TCH, date of approval: 12 December 2013).

### 4.2. Extraction of RNA and Purification of mRNA

The venom gland tissue was homogenized in a 1 ml glass homogenizer with TRIzol solution (Invitrogen, Calsbad, CA, USA) aseptically. Then, 20% chloroform was added, and the sample was centrifuged and treated with RNA-free DNAase I to separate RNA from the cellular debris and residual DNA. The separated RNA was then pelleted using isopropyl alcohol and washed with 75% ethanol. The polyadenylated mRNA (poly(A)^+^ mRNA) was purified with oligo (dT) magnetic beads from 20 μg of total RNA, as per manufacturer’s instructions (Illumina, San Diego, CA, USA). The quality of the purified RNA was assessed immediately using the Agilent 2100 Bioanalyzer (Agilent Technologies, Waldbronn, Germany). The RNA integrity number (RIN) of the sample was determined to be 8.6, indicating that the RNA was in good condition for downstream transcriptomic analysis.

### 4.3. Construction of cDNA Library and Sequencing

Construction of the cDNA library was performed with the previously enriched poly(A)^+^ mRNA isolated from of the total venom-gland RNA. Following purification, the isolated mRNA was fragmented into short fragments by standard buffers containing divalent cations (Zn^2+^) to mediate the production of homogeneous fragments [65]. The fragments were then served as templates for cDNA synthesis. The first strand of cDNA was synthesized with random hexamer-primer (N6), followed by second strand cDNA synthesis using second strand buffers, RNase H, dNTPs, and DNA polymerase I. Purification of these short fragments was performed with QIAquick PCR extraction kit (Qiagen, Valencia, CA, USA). Purified short fragments are then dissolved with EB buffer for end repair, and the addition of a single adenine nucleotide to assist in the subsequent ligation of Illumina adaptors that contain a single thymine (T) base overhang at the 3’ ends. After the sequencing adaptors were ligated, these short fragments of cDNA were amplified via polymerase chain reaction (PCR) under the following conditions: an initial denaturation at 95 °C for 10 min, followed by 95 °C denaturation 30 s, 60 °C annealing 60 s, and 72 °C extension 60 s, for 40 cycles. The PCR products were then subjected to electrophoresis on 1.5–2% TAE (Tris base, acetic acid and EDTA) agarose gel. From the gel, fragments of 200–700 nucleotides were selected as templates for PCR amplification. PCR was carried out as follows: an initial denaturation at 95 °C for 12 min, 20 cycles of denaturation at 95 °C for 30 s, annealing at 60 °C for 30 s, and polymerization at 72 °C for 40 s, and a final extension cycle 72 °C for 5 min. The qualification and quantification of sample library were accessed with ABI StepOnePlus Real-time PCR system (Applied Biosystem, Foster, CA, USA) and Agilent 2100 Bioanalyzer (Agilent Technologies, Waldbronn, Germany). Sequencing of the amplified samples library was achieved in a single lane on the Illumina HiSeq^TM^ 2000 platform (Illumina, San Diego, CA, USA) with a 100-base pair, pair-end reads. 

### 4.4. Filtration of Raw Sequence Data

Sequence data generated from Illumina HiSeq^TM^ 2000 were transformed into raw reads, stored in a FASTQ format. Prior to transcriptome assembly, filtration of the raw sequencing reads was performed to generate clean reads. This involved removal of low quality reads which possessed more than 5% ambiguous nucleotides, reads containing more than 20% bases with quality score of Q <10 and/or those containing adaptor sequences, using an in-house validated filtering program (Filter_fq, BGI, Yantian, ShenZhen, China).

### 4.5. Assembly of De Novo Transcriptome

The de novo ‘shot-gun’ transcriptome assembly was performed with Trinity, a short-reads assembly program [66]. The three independent software modules, i.e., Inchworm, Chrysalis, and Butterfly, constitute the Trinity program used to process the large volumes of RNA-seq reads based on De Bruijn graph construction that began by aligning *k*-mers (*k* = 25). Reads with a certain length of overlapping were joined to form linear contigs. By referring to pair-end reads, contigs of the same transcripts and the distance between them were determined. Contigs were then categorized into clusters, with each cluster possessing their own set of de Bruijn graphs as a representation of the transcriptional complexity of a given gene or locus. Full-length transcripts were obtained through individual processing of each generated graph, for alternatively spliced isoforms and to tease transcripts corresponding to paralogous genes. For quality control assessment, output statistics was performed with determining the Q20 percentage that serves as a benchmark. Unigenes and contigs were categorized and illustrated in Figure 1. The process was done based on the equation shown below:

Total Clean Nucleotide=(Total Clean Reads 1 x Read 1 size)+(Total Clean Reads 2 x Reads 2 size)

### 4.6. Clustering of Transcripts

Transcript sequences that were generated from Trinity were labeled as Unigenes. Unigenes were further processed for sequence splicing and removal of redundant reads with TGI clustering tools (TGICL), version 2.1, to obtain long non-redundant (NR) transcripts at the longest possible length. The transcripts then underwent family clustering, to classify into: (1) singletons, ID with a prefix of Unigene (2) clusters, ID with the prefix CL and cluster ID are at the end as contigs. In each cluster, there were several transcripts possessing sequence similarities of more than 70% (containing various contigs); whereas singleton ‘Unigenes’ are lack of overlapping with other fragments at a given stringency. 

Next, Unigenes were aligned with BLASTx protein database exclusive to NCBI non-redundant database (NR), with significance cut-off value E <10^−5^ High ranked proteins were then referred to determine the coding region of the Unigenes, followed by translation into amino acid sequences with the standard codon table, hence, both nucleotide sequences (both 5’ and 3’ end) and amino sequences of the Unigene-coding regions were obtained. Several procedures were performed for housekeeping and standardization of the data: redundancy was removed with the selection of the longest sequence present in each cluster as a transcript. Scaffold lengths were extended based on the overlapping sequences applying Phrap assembler (release 23.0) (http://www.phrap.org). The length distributions of the contigs, scaffolds and Unigenes were calculated. For assembly success, the N50 length statistics was set at N50 >500.

### 4.7. Quantifying the Expression Annotation of Transcripts

The FPKM method of Mortazavi et al. [67] was adapted to determine transcript abundances for the identified genes. The data was computed using RNA-seq with expectation maximization (RSEM) tool incorporated in Trinity, the assembly program according to the formula stated below:

FPKM of gene A=106CN L103

The FPKM method was applied to eliminate the influence of different gene lengths and sequencing discrepancy on the calculation of gene expression. FPKM is defined as the expression of gene A; *C* is the number of fragments (i.e., reads) that are uniquely aligned to the gene A; *N* is the total number of fragments (i.e., reads) that are uniquely aligned to all genes; L is the base number in the coding sequence (CDS) of gene A.

### 4.8. Determination of Functional Annotation of Transcripts

Proteins derived in the process were aligned with the aid of BLASTx to obtain the most resembling sequences present in NR non-redundant protein database to provide proteins functional annotation. The annotation of transcripts provides information about mRNA expression (shown above) and the putative identity of the genes as illustrated in the Supplementary File S1.

### 4.9. Classification of Venom-Gland Transcripts Based on Toxinology

The transcripts (Unigenes) obtained in the preceding step were filtered to remove transcripts with FPKM <1. Post-filteration transcripts with expression >1 FPKM were broadly categorized into “toxins”, “non-toxins”, and “unidentified”. “Toxin” transcripts were derived by the search-and-find method for specific toxin-keywords against the annotations of the transcripts. Their identities were further validated by subjecting the amino acid sequences to BLASTp (Basic Local Alignment Search Tool-Protein) search in UniProt (Universal Protein Resource Knowledgebase) databank exclusively set to the taxonomy of Serpentes (as of 9th February 2018), applying the lowest E-score value and the highest similarity percentage for annotation purposes. Toxin transcripts were then categorized according to their respective protein families (Supplementary File S2). Toxin transcripts with high similarity to Viperidae (vipers and pit vipers), and FPKM <10 were excluded from the analysis for the possibility of trace contamination. The transcripts of cellular proteins and house-keeping genes were categorized as the “non-toxin” group, whereas those transcripts that could not be identified were classified as “unidentified”.

Relative gene expression (FPKM) of each group was summed and expressed in percentage over the total expression, followed by determining gene expression redundancy for genes by dividing the total transcript FPKM of each group by the total number of transcripts of their respective group. High redundancy indicates high expression level of gene group.

### 4.10. Multiple Sequence Alignment and Phylogenetic Tree Construction

Selected transcripts obtained in the current study were subjected for multiple sequence alignment. The amino acid sequences obtained from the current study were aligned with toxin sequences retrieved from the Elapidae database in UniprotKB depository (http://www.uniprot.org/). Multiple sequence alignment was achieved with Jalview software (version 2.10.5) [68] and MUSCLE (Multiple Sequence Comparison by Log-Expectation) [69]. Selected transcript sequences were also subjected to phylogenetic tree construction using Mega X (version 10.0.5), and the default statistical method of Maximum Likelihood with bootstrap value of 100 [70].

### 4.11. Supporting Data

Sequence data from the venom-gland transcriptome of Malaysian *Naja sumatrana* (NS-M) has been deposited in National Centre for Biotechnology Information (NCBI) Sequence Read Achieve (https://trace.ncbi.nlm.nih.gov/Traces/sra/sra.cgi) under submission ID: SUB4976990 (https://submit.ncbi.nlm.nih.gov/subs/sra/SUB4976990).

## Figures and Tables

**Table 1:** Output statistics of *de novo* assembly of *Naja sumatrana* venom-gland transcriptome using Illumina HiSeq 2000 sequencing.

Parameter	Sequencing Output
Total raw reads	47,494,560
Total clean reads	46,878,172
Contigs created	148,475
Q20 percentage	97.94%
*N* percentage	0.00%
GC percentage	44.16%
Unigenes/transcripts assembled	75,387
Number of transcripts (FPKM > 1)	55,386
Unidentified	Abundance
Number of transcripts	35,449
Clean reads	123,432.1986
Total FPKM percentage (%)	7.95%
Non-toxin	Abundance
Number of transcripts	19,877
Clean reads	199,393.4726
Total FPKM percentage (%)	12.84%
Toxin	Abundance
Number of transcripts	60
Clean reads	1,230,548.6634
Total FPKM percentage (%)	79.22%

**Table 2:** Full-length toxin transcripts derived from the venom-gland transcriptome of Malaysian *Naja sumatrana* (NS-M).

Protein Family/Protein ID		Annotated Accession	Species	Amino Acid Chain	Mature Chain of Accession ID	Coverage (Mature Chain)	Coverage Percentage (%)
**Three-finger toxin**							
NSM_FTX01	Cytotoxin 2a	Q9PST4	*N. sputatrix*	81	81	1–81	100
NSM_FTX02	Neurotoxin homolog NL1	Q9DEQ3	*N. atra*	81 ^a^	86	6–86	93
NSM_FTX06	Long neurotoxin 7	O42257	*N. sputatrix*	89	90	2–90	98
NSM_FTX07	Alpha-neurotoxin NTX-4	O57327	*N. sputatrix*	83	83	1–83	100
**Phospholipase A_2_**							
NSM_PLA01	Acidic phospholipase A_2_ C	Q92086	*N. sputatrix*	146	146	1–146	100 ^b^
**Cysteine-rich secretory protein**							
NSM_CRP01	Cysteine-rich venom protein natrin-1	Q7T1K6	*N. atra*	239 ^a^	239	1–239	100 ^b^
**Nerve growth factor**							
NSM_NGF01	Venom nerve growth factor 2	Q5YF89	*N. sputatrix*	246	241	1–241	100 ^b^
**Vespryn**							
NSM_VES01	Ohanin	P83234	*O. hannah*	190 ^a^	190	1–190	100 ^b^
**C-type/lectin-like protein**							
NSM_SCL01	C-type lectin BfL-1	Q90WI8	*B. fasciatus*	158 ^a^	158	1–158	100 ^b^
**Snake venom metalloproteinase**							
NSM_SMP07	Zinc metalloproteinase-disintegrin-like kaouthiagin-like	D3TTC1	*N. atra*	593 ^a^	593	1–593	100 ^b^
NSM_SMP08	Zinc metalloproteinase-disintegrin-like atrase-B	D6PXE8	*N. atra*	613 ^a^	593	1–593	100 ^b^
**5’ nucleotidase**							
NSM_NUC01	Ecto-5’-nucleotidase 1	U3FYP9	*M. fulvius*	569 ^a^	574	1–569	99
**L-amino-acid oxidase**							
NSM_LAO01	L-amino-acid oxidase	A8QL58	*N. atra*	514 ^a^	449	1–449	100 ^b^
**Cobra venom factor**							
NSM_CVF01	Cobra venom factor	Q91132	*N. kaouthia*	1646 ^a^	1642	1–1642	100 ^b^
**Kunitz-type serine protease inhibitor**							
NSM_KPI01	Putative Kunitz-type serine protease inhibitor	B2BS84	*A. labialis*	249	252	1–252	98
NSM_KPI02	Kunitz-type protease inhibitor	U3FZD6	*M. fulvius*	513	511	1–511	100 ^b^
**Aminopeptidase**							
NSM_AP01	Aminopeptidase	U3FZS8	*M. fulvius*	1000	993	1–993	100 ^b^
**Phospholipase B**							
NSM_PLB01	Phospholipase-B 81	F8J2D3	*D. coronoides*	553 ^a^	553	1–553	100 ^b^
**Vascular endothelial growth factor**							
NSM_VGF01	Vascular endothelial growth factor 2	U3FAK1	*M. fulvius*	421	421	1–421	100 ^b^
**Dipeptidylpeptidase IV**							
NSM_DPP01	Dipeptidyl peptidase 4	V8P9G9	*O. hannah*	753	754	1–753	99
**Neprilysin**							
NSM_NP01	Neprilysin	A0A0B8RU83	*B. irregularis*	750	750	1–750	100 ^b^
**Hyaluronidase**							
NSM_HY01	Hyaluronidase	A0A194APD1	*M. tener*	449	447	1–447	100 ^b^
NSM_HY02	Hyaluronidase	A0A194APD1	*M. tener*	449	447	1–447	100 ^b^
NSM_HY03	Hyaluronidase	A0A194APD1	*M. tener*	449	447	1–447	100 ^b^
NSM_HY04	Hyaluronidase	A0A194APD1	*M. tener*	449	447	1–447	100 ^b^
**Phosphodiesterase**							
NSM_PDE01	Snake venom phosphodiesterase	A0A2D0TC04	*N. atra*	850	830	1–830	100 ^b^
NSM_PDE02	Snake venom phosphodiesterase	A0A2D0TC04	*N. atra*	848	830	1–830	100 ^b^
**Snake venom serine protease**							
NSM_SSP01	Serine protease HTRA1	A0A0B8RTL3	*B. irregularis*	471	489	18–488	96
NSM_SSP02	Serine protease 23	V8N8N4	*O. hannah*	365	372	8–372	98

Notes: *A.*, *Austrelaps*; *B.*, *Boiga*/*Bungarus*; *C.*, *Crotalus*; *D.*, *Drysdalia*; *M.*, *Micrurus*; *N.*, *Naja*; *O.*, *Ophiophagus*; and *T.*, *Trimeresurus*. ^a^ Novel sequence reported in *Naja sumatrana*; ^b^ Protein sequence not identical to the annotated sequence.

**Table 3:** Overview of families and subtypes of toxin genes in the venom-gland transcriptome of Malaysian *Naja sumatrana* (NS-M).

Protein Family/Protein Subtype	Accession/Species	Transcript Abundance % ^a^
**Three-finger toxin (3FTx)**		91.11 (10)
*S-3FTx*		*73.14 (5)*
Cytotoxin 2a	Q9PST4 (*N. sputatrix*)	72.83 (1) ^b^
Neurotoxin homolog NL1	Q9DEQ3 (*N. atra*)	0.21 (1) ^b^
Cardiotoxin 7	Q91996 (*N. atra*)	0.10 (2)
Muscarinic toxin-like protein 1	P82462 (*N. kaouthia*)	0.00 (1)
*L-3FTx*		*8.23 (4)*
Long neurotoxin 7	O42257 (*N. sputatrix*)	4.91 (1) ^b^
Alpha-neurotoxin NTX-4	O57327 (*N. sputatrix*)	3.30 (1) ^b^
Long neurotoxin-like OH-31	Q53B55 (*O. hannah*)	0.01 (2)
*UC-3FTx*		*9.74 (1)*
Weak neurotoxin 5	O42255 (*N. sputatrix*)	9.74 (1)
**Phospholipase A_2_ (PLA_2_)**		7.42 (2)
Acidic phospholipase A_2_ C	Q92086 (*N. sputatrix*)	7.39 (1) ^b^
Phospholipase A_2_ GL16-1	Q8JFB2 (*L. semifasciata*)	0.03 (1)
**Cysteine-rich secretory protein (CRISP)**		0.33 (4)
Cysteine-rich venom protein natrin-1	Q7T1K6 (*N. atra*)	0.32 (1) ^b^
Cysteine-rich venom protein natrin-2	Q7ZZN8 (*N. atra*)	0.01 (2)
Cysteine-rich venom protein kaouthin-2	P84808 (*N. kaouthia*)	0.01 (1)
**Nerve growth factor (NGF)**		0.31 (2)
Venom nerve growth factor 2	Q5YF89 (*N. sputatrix*)	0.31 (1) ^b^
Venom nerve growth factor 1	Q5YF90 (*N. sputatrix*)	0.00 (1)
**Vespryn (VES)**		0.25 (1)
Thaicobrin	P83234 (*O. hannah*)	0.25 (1) ^b^
**Snake venom C-type/lectin-like protein (Snaclec)**		0.22 (2)
C-type lectin BfL-1	Q90WI8 (*B. fasciatus*)	0.22 (1) ^b^
C-type lectin BfL-2	Q90WI7 (*B. fasciatus*)	0.00 (1)
**Snake venom metalloproteinase (SVMP)**		0.17 (10)
Snake venom metalloproteinase-disintegrin-like morcarhagin	Q10749 (*N. mossambica*)	0.03 (1)
Zinc metalloproteinase-disintegrin-like cobrin	Q9PVK7 (*N. kaouthia*)	0.07 (3)
Carinatease-1	B5KFV1 (*Tr. carinatus*)	0.02 (1)
Zinc metalloproteinase-disintegrin-like atrase B	D6PXE8 (*N. atra*)	0.03 (3) ^b^
Zinc metalloproteinase-disintegrin-like kaouthiagin-like	D3TTC1 (*N. atra*)	0.01 (1) ^b^
Zinc metalloproteinase-disintegrin-like atrase A	D5LMJ3 (*N. atra*)	0.01 (1)
**5’ nucleotidase (5’ NUC)**		0.05 (2)
Ecto-5’-nucleotidase 1	U3FYP9 (*M. fulvius*)	0.05 (1) ^b^
5’ nucleotidase	A0A024AXW5 (*M. ikaheca*)	0.00 (1)
**Natriuretic peptide (NP)**		0.05 (2)
Natriuretic peptide Na-NP	D9IX97 (*N. atra*)	0.05 (2)
**L-amino acid oxidase (LAAO)**		0.02 (1)
L-amino-acid oxidase	A8QL58 (*N. atra*)	0.02 (1) ^b^
**Cobra venom factor (CVF)**		0.02 (3)
Cobra venom factor	Q91132 (*N. kaouthia*)	0.02 (3) ^b^
**Kunitz-type serine protease inhibitor (KSPI)**		0.01 (2)
Putative Kunitz-type serine protease inhibitor	B2BS84 (*A. labialis*)	0.01 (1) ^b^
Kunitz-type protease inhibitor	U3FZD6 (*M. fulvius*)	0.00 (1) ^b^
**Aminopeptidase (AP)**		0.01 (1)
Aminopeptidase	U3FZS8 (*M. fulvius*)	0.01 (1) ^b^
**Phospholipase B (PLB)**		0.01 (1)
Phospholipase-B 81	F8J2D3 (*D. coronoides*)	0.01 (1) ^b^
**Cystatin**		0.01 (5)
Cystatin	V8NX38 (*O. hannah*)	0.00 (1)
Cystatin	A0A098LYB6 (*O. aestivus*)	0.00 (4)
**Vascular endothelial growth factor (VEGF)**		0.01 (2)
Vascular endothelial growth factor 2	U3FAK1 (*M. fulvius*)	0.01 (1) ^b^
Vascular endothelial growth factor A	A0A098LYD7 (*O. aestivus*)	0.00 (1)
**Dipeptidylpeptidase IV (DPP IV)**		0.00 (1)
Dipeptidyl peptidase 4	V8P9G9 (*O. hannah*)	0.00 (1) ^b^
**Neprilysin**		0.00 (1)
Neprilysin	A0A0B8RU83 (*B. irregularis*)	0.00 (1) ^b^
**Hyaluronidase (HY)**		0.00 (4)
Hyaluronidase	A0A194APD1 (*M. tener*)	0.00 (4) ^b^
**Phosphodiesterase (PDE)**		0.00 (2)
Snake venom phosphodiesterase	A0A2D0TC04 (*N. atra*)	0.00 (2) ^b^
**Snake venom serine protease (SVSP)**		0.00 (2)
Serine protease HTRA1	A0A0B8RTL3 (*B. irregularis*)	0.00 (1) ^b^
Serine protease 23	V8N8N4 (*O. hannah*)	0.00 (1) ^b^

*A.*, *Agkistrodon*/*Austrelaps*; *B.*, *Boiga*/*Bothrops*/*Bungarus*; *C.*, *Crotalus*; *D.*, *Drydalia*; *E.*, *Elaphe*; *L.*, *Laticauda*; *M.*, *Micrurus*/*Micropechis*; *N.*, *Naja*; *O.*, *Opheodrys*/*Ophiophagus*; *T.*, *Trimeresurus*; and *Tr.*, *Tropidechis*. ^a^ Transcript expression in percentage (%) based on FPKM (fragments per kilobase of exon model per million mapped reads). Number in bracket refers to the number of non-redundant transcripts in the toxin family. ^b^ Full-length transcript with more than 90% length coverage of the annotated sequence.

## Supplementary Materials

The following are available online at http://www.mdpi.com/2072-6651/11/2/104/s1, Supplementary File S1: Categorization of venom-gland transcripts from Malaysian *Naja sumatrana*; Supplementary File S2: Venom-gland transcriptomic analysis of toxins from Malaysian *Naja sumatrana*.
